# Human milk microbial species are associated with infant head-circumference during early and late lactation in Guatemalan mother-infant dyads

**DOI:** 10.3389/fmicb.2022.908845

**Published:** 2022-11-16

**Authors:** Tamara T. Ajeeb, Emmanuel Gonzalez, Noel W. Solomons, Kristine G. Koski

**Affiliations:** ^1^School of Human Nutrition, McGill University, Montréal, QC, Canada; ^2^Department of Clinical Nutrition, College of Applied Medical Sciences, Umm Al-Qura University, Makkah, Saudi Arabia; ^3^Canadian Centre for Computational Genomics, McGill Genome Centre, Montréal, QC, Canada; ^4^Department of Human Genetics, McGill University, Montréal, QC, Canada; ^5^Gerald Bronfman Department of Oncology, McGill University, Montréal, QC, Canada; ^6^Center for Studies of Sensory Impairment, Aging and Metabolism (CeSSIAM), Guatemala City, Guatemala

**Keywords:** human milk, lactation, Guatemala, Indigenous population, infant growth, head-circumference, microbiome, 16S rRNA gene

## Abstract

Human milk contains abundant commensal bacteria that colonize and establish the infant’s gut microbiome but the association between the milk microbiome and head circumference during infancy has not been explored. For this cross-sectional study, head-circumference-for-age-z-scores (HCAZ) of vaginally delivered breastfed infants were collected from 62 unrelated *Mam*-Mayan mothers living in eight remote rural communities in the Western Highlands of Guatemala during two stages of lactation, ‘early’ (6–46 days postpartum, *n* = 29) or ‘late’ (109–184 days postpartum, *n* = 33). At each stage of lactation, infants were divided into HCAZ ≥ −1 SD (early: *n* = 18; late: *n* = 14) and HCAZ < −1 SD (early: *n* = 11; late: *n* = 19). Milk microbiome communities were assessed using 16S ribosomal RNA gene sequencing and DESeq2 was used to compare the differential abundance (DA) of human milk microbiota with infant HCAZ subgroups at both stages of lactations. A total of 503 ESVs annotated 256 putative species across the 64 human milk samples. Alpha-diversity using Chao index uncovered a difference in microbial community richness between HCAZ ≥ −1 SD and HCAZ < −1 SD groups at late lactation (*p* = 0.045) but not at early lactation. In contrast, Canonical Analysis of Principal Coordinates identified significant differences between HCAZ ≥ −1 SD and HCAZ < −1 SD at both stages of lactation (*p* = 0.003); moreover, 26 milk microbial taxa differed in relative abundance (FDR < 0.05) between HCAZ ≥ −1 SD and HCAZ < −1 SD, with 13 differentially abundant at each lactation stage. Most species in the HCAZ ≥ −1 SD group were *Streptococcus* species from the Firmicutes phylum which are considered human colonizers associated with human milk whereas the HCAZ < −1 SD group at late lactation had more differentially abundant taxa associated with environmentally and ‘potentially opportunistic’ species belonging to the Actinobacteria genus. These findings suggest possible associations between brain growth of breastfed infants and the milk microbiome during lactation. Importantly, these data provide the first evidence of cross talk between the human milk microbiome and the infant brain that requires further investigation.

## Introduction

Human milk is a complex dynamic fluid that contains both nutrients and non-nutritive bioactive factors to meet the nutritional and developmental requirements of the growing infant (Petherick, 2010; Walker, 2010; Andreas et al., 2015). It also contains a diverse community of commensal and potential probiotic bacteria that can inoculate the infant gastrointestinal tract (Fernández et al., 2013; McGuire and McGuire, 2017; Boudry et al., 2021) and are associated with infant health (McGuire and McGuire, 2015). Microbiota are involved in metabolic pathways that contribute to infant growth, such as harvesting energy (Cani and Delzenne, 2009), synthesizing vitamins (Kho and Lal, 2018), regulating the immune system, and development and maturation of both the enteral- and central nervous system through the gut-brain and brain-gut axes (Carabotti et al., 2015).

Metagenomic sequencing studies are advancing the characterization of the diverse bacterial species in human milk. The latest systematic reviews of studies of the human milk microbiome using culture independent methods and different 16S rRNA variable regions concluded that *Streptococci* and *Staphylococci* are the most predominant genera in human milk (Fitzstevens et al., 2017; Sakwinska and Bosco, 2019). Furthermore, the milk microbiome ecosystem appears more diverse in terms of bacterial species but more similar among samples compared to infants’ fecal or oral ecosystems. This phylogenetic structure revealed the dominance of *Streptococcaceae*, with *Streptococcus* being the dominant genus in the milk ecosystem (Biagi et al., 2017).

Evidence also supports the link between gut microbiota and the development of the nervous system that involves bidirectional communication of neuronal pathways essential for neurological development and brain growth (Rogers et al., 2016; Niccolai et al., 2019). Human milk is one of the first and continuous inoculators of the infant gut microbiota. Although human milk microbiota is a critical contributor to establishing infant gut microbiota (Fernández et al., 2013; McGuire and McGuire, 2017), and the infant consumes up to 10 million microbiota per day (Gomez-Gallego et al., 2016), the association between human milk microbiome and infant head circumference, as a measure of brain growth (Cheong et al., 2008), is yet to be explored.

The neonatal period is characterized by rapid brain growth (Huttenlocher and Dabholkar, 1997; Knickmeyer et al., 2008; Tau and Peterson, 2010) that coincides with the maturation of the gut microbiota (Yatsunenko et al., 2012). Investigations have revealed that the gut microbiota is involved in the modulation of brain development during infancy and neonatal period through a complex bidirectional communication microbiota-brain axis network (Borre et al., 2014; Diaz Heijtz, 2016; Gonzalez-Santana and Heijtz, 2020), suggesting early life as a sensitive period for microbiota-gut-brain interactions (Yatsunenko et al., 2012; Cryan et al., 2019). Germ-free mice studies have shown that *Lactobacillus* families communicate with the brain through the vagus nerve (Buffington et al., 2016; Sgritta et al., 2019), which may be one of the most direct routes though which microbiota communicate with the brain (Fülling et al., 2019), but the precise mechanism is not fully understood. Another potential microbiota-brain-axis mechanism is the gut hormone signaling (Heijtz et al., 2011) whereby the hypothalamic–pituitary–adrenal axis through release of adrenocorticotropic hormone and glucocorticoids modulate the intestinal epithelial barrier and immune responses and subsequently gut microbiota composition (Cryan et al., 2019; Gonzalez-Santana and Heijtz, 2020). However, the precise mechanisms and signaling pathways involved in the microbiota-gut-brain interaction of the developing brain during early life are not fully understood. Establishing associations between microbiota and early life brain development might have important implications for early brain development.

In this cross-sectional study, we aimed to compare the association of the human milk microbiome of breastfeeding Guatemalan mothers with their infants’ head-circumference-for-age-z-score (HCAZ) as a proxy measure of brain growth (Cheong et al., 2008). We compared the diversity and differential abundance (DA) of the human milk microbiome between infants with HCAZ ≥ −1 SD vs. HCAZ < −1 SD during both early lactation (6–46 days postpartum) and late lactation (109–184 days postpartum). We also aimed to identify milk microbiome species that are differentially abundant between infant HCAZ subgroups, to better understand the association between the human milk microbiome and brain development in early life as an important growth variable that may influence the neurodevelopmental outcomes in lactating mother-infant dyads living in the remote Western Highlands of Guatemala.

## Materials and methods

### Study setting, recruitment and ethics

This was a cross-sectional study that was conducted in eight rural *Mam*-Mayan communities of the Western Highland departments of Quetzaltenango between June 2012 and January 2013 (Chomat et al., 2015).

The *Mam*-Mayan community constitutes the fourth largest Mayan population in Guatemala) (Instituto Nacional de Estadística, 2003). Even though mothers were recruited from eight distinct remote communities to minimize the possibility of exchanging microbes among one another, these eight Mam-Mayan communities were characterized by dispersed houses. These communities had high rates of poverty (68% extreme poverty and 19% poverty) and food insecurity (62%) (Escobar, 2009; Chomat et al., 2015). *Mam*-Mayan are known to comply with WHO recommendations to exclusively or predominantly breastfeed for the first 6 months of the infant’s life (Wren et al., 2015; World Health Organization [WHO], 2017).

Lactating mothers were recruited by community health workers using a participatory action research approach (Cargo and Mercer, 2008). Recruitment methods included home visits, loudspeaker announcements, and word-of-mouth invitations (Chomat et al., 2015). All mothers delivered vaginally and nearly 100% of mothers exclusively or predominantly breastfed their infants for 6 months. Mothers with infants < 4 days or with milk volumes insufficient for analysis, mothers treated with antibiotics or who had a non-singleton birth were excluded (Wren et al., 2015). A previous study in our population revealed that sub-clinical mastitis was associated with a low head circumference (Wren-Atilola et al., 2019). Thus, mothers with sub-clinical mastitis (milk Na:K > 0.6) were also excluded. Inclusion criteria were mother-infant dyads aged 6–46 days and 109–184 days postpartum.

The study was a collaboration between McGill University and the Center for Studies of Sensory Impairment, Aging, and Metabolism, a research organization based in Guatemala. Both ethics boards approved the study. In addition, permissions were obtained from community leaders and the local authorities of the Ministry of Health. A fully informed written consent (thumbprint if unable to sign) was obtained from women if they wished to participate, and all mothers were informed of their rights to withdraw from the study at any time.

### Human milk sample collection

Milk samples were collected during the day in a 3-h time window between 9 a.m. and 12 p.m. from a single, unilateral breast milk sample, from the breast that was not last used to feed the infant ensuring that foremilk was collected from all mothers, which can minimize the variation in milk composition. Only manual expression was used for milk collection, which exclusively involves hand expression of breast milk, without the use of a breast pump. Milk samples were collected by a trained midwife, who used hand sanitizer before and after collection (Wren et al., 2015). The nipple and areola of the breast, not recently used for breastfeeding, were cleaned with 70% ethanol prior to sample collection into sterile 60 ml plastic vials and stored on ice immediately. In the field laboratory, milk samples were partitioned, stored at −30°C before being shipped to McGill University in two separate shipments (Li et al., 2016).

### Infant anthropometry

According to standardized procedures and as described in the detailed methodology previously published, infant measurements were taken by two trained Guatemalan nutritionists (Chomat et al., 2015). In brief, infant recumbent supine length (cm) was measured thrice using an infantometer (SECA 210) and recorded to the nearest 0.5 cm. The mean was calculated and considered the final value. Infant weight (kg) was measured using a digital infant scale (SECA 354) and rounded to the nearest 100 g. Head circumference (cm) was measured thrice using a head circumference baby band (SECA 212). All infant anthropometric measures were completed on the same day of milk sample collection. Infant age was either calculated using date of birth recorded on the maternal health card or obtained from the mother in the absence of a health card. Infant length-for-age z-score (LAZ), weight-for-age z-score (WAZ), and head circumference for age z-score (HCAZ) were calculated as indicators of infant growth status using the World Health Organization Anthro software. To assess head-circumference (HC), infant HCAZ was calculated as an indicator of brain development at early and late lactation using the World Health Organization Anthro software (3.1) (World Health Organization [WHO], 2006). Due to the low prevalence of microcephaly (HCAZ < −2 SD) in our population, we used the cut-off point of HCAZ < −1 SD to categorize the infant with low HC. Based on HCAZ, infants were classified into two groups at two stages of lactation: head-circumference HCAZ ≥ −1 SD (*n* = 18) and low or mild impairment in head-circumference HCAZ < −1 SD (*n* = 11) in early lactation and HCAZ ≥ −1 SD (*n* = 14) and HCAZ < −1 SD (*n* = 19) in late lactation.

### 16S rRNA gene amplification and sequencing

A DNeasy Blood and Tissue mini kit from Qiagen was used to extract DNA from 1 ml of milk in accordance with the manufacturer’s protocol by Genome Quebec. For PCR, the universal eubacteria primers 27F/533R (27F: AGAGTTTGATCCTGGCTCAG, 533R: TTACCGCGGCTGCTGGCAC) were used for the amplification of the variable regions V1–V3 consisting of ∼526 bp based on the *Escherichia coli* 16S rRNA gene (Cabrera-Rubio et al., 2012; Mediano et al., 2017; Lackey et al., 2019). The primers were chosen because of their high coverage of most genera currently considered “core” in human milk, including the genus *Cutibacterium* (Hunt et al., 2011; Jost et al., 2013). Genome Quebec conducted amplification at McGill University. The amplification conditions have been previously described (Gonzalez et al., 2021).

### Microbial data processing

Genome Quebec at McGill University conducted the amplification, and sequencing was performed using Illumina MiSeq. The amplification process was previously described (Gonzalez et al., 2021). The ANCHOR pipeline was chosen for amplicon sequence processing. The platform was designed for improved species-level microbial identification using direct paired-end sequences, which helps to substantially improve the sequence resolution of 16S rRNA gene amplification data; furthermore, it uses integrated multiple-reference database annotation to enhance the interpretation of complex, non-reference microbiomes (Gonzalez et al., 2019). In brief, Mothur (Schloss et al., 2009) was used to align and dereplicate sequences. The databases NCBI 16S rRNA RefSeq, NCBI non-redundant nucleotide, SILVA, and the Ribosomal Database Project were used to annotate ESVs using BLASTn with criteria of > 99% for identity and coverage. Due to the high standard of curation, priority was given to NCBI 16S rRNA RefSeq, when 100% identity and coverage hits returned across multiple databases. Amplicons with low counts (<36) were binned to high-count ESVs at a low threshold of > 98% identity/coverage. Taxonomy annotation, particularly species calls, should be considered putative even when sharing 100% sequence identity to a single species due to database errors.

### Bioinformatics

To address sparsity issues, only ESVs with at least 3 counts in 3 different samples from a same group of comparison were conserved. No data normalization was performed for alpha-diversity. rlog normalization (rlog function in Phyloseq R package) was used for data transformation in beta-diversity analysis (Gonzalez et al., 2021).

Alpha-diversity evaluates human milk microbiome communities within samples, which was measured using Phyloseq R package with R Studio software (version 1.4.1106) (McMurdie and Holmes, 2013). Six different alpha-diversity metrics were used to estimate and compare microbial richness within samples (Observed, Chao-1, Shannon, Simpson, Inverse Simpson, and Fisher). And thereafter comparing microbial richness between HCAZ infant groups was done using *t*-tests on the richness measures. ACE and Chao-1 were used to account for taxonomies that were undetected due to low abundance. We used Observed to calculate the total number of unique ESVs per sample. To account for equitability in sample distribution Shannon index was used, and for the species dominance we used Simpson. Fisher was used to account for uncertainty in richness estimations.

Beta-diversity was determined to evaluate differences in human milk microbiome communities between the HCAZ ≥ −1 SD and HCAZ < −1 SD at both early and late lactation. To evaluate and visualize the differences between the four different groups, constrained ordination was employed based on Bray-Curtis dissimilarity computed on rlog-transformed counts using Canonical Analysis of Principal Coordinates (CAP). ANOVA-like permutation statistical test was used for the significance of the different constraints. DESeq2 procedure (Love et al., 2014) was used to evaluate differentially abundant taxonomic units between HCAZ ≥ −1 SD and HCAZ < −1 SD groups to identify statistical differences between microbial communities. DESeq2 identifies significant differences between groups while considering the library size. Difference in abundance between microbial communities tested with a false discovery rate (FDR < 0.05) were considered significant.

## Results

### Characterization of Guatemalan mother-infant dyads

Maternal and infant characteristics are summarized in Table 1. Maternal characteristics which were divided by infant HCAZ ≥ −1 SD and HCAZ < −1 SD, revealed no significant differences in either early or late lactation for age, height, weight, BMI, parity, or breastfeeding practices.

**TABLE 1:** Population characteristics of mothers and infants at 2 stages of lactation (x̄ ± SD or %).

Characteristics	Early lactation			Late lactation		
	HCAZ ≥ −1 SD*^a^*	HCAZ < −1 SD*^a^*	*P*-value^b^	HCAZ ≥ −1 SD*^a^*	HCAZ < −1 SD*^a^*	*P*-value^b^
*n*	18	11		14	19	
**Maternal**						
Age, yrs	23 ± 6	23 ± 5	>0.9	23 ± 6	22 ± 8	0.9
Height, cm	146 ± 5	146 ± 5	0.6	147 ± 5	148 ± 5	>0.9
Weight, kg	51 ± 7	50 ± 6	0.5	53 ± 10	51 ± 8	0.5
BMI, kg/m^2^	23.7 ± 2.3	23.4 ± 3.9	0.8	24.5 ± 4.1	23.4 ± 3.3	0.6
Parity, %			0.9			0.3
Primiparous	44	36		64	33	
Multiparous	56	64		35	67	
Breastfeeding Practices, %			0.8			0.2
Exclusive	50	55		43	53	
Predominant	50	45		14	32	
Mixed	−	−		43	16	
SCM, Na:K	0.42 ± 0.08	0.40 ± 0.06	0.2	0.38 ± 0.12	0.40 ± 0.07	0.9
**Infant**						
Age, d	23 ± 10	23 ± 12	0.7	147 ± 17	139 ± 22	0.2
Sex, %			0.7			0.8
Male	56	64		57	53	
Female	44	36		43	47	
Head-Circumference, cm	36.09 ± 1.03	34.51 ± 1.87	0.01	41.55 ± 0.97	39.28 ± 1.49	<0.001
HCAZ	−0.17 ± 0.53	−1.43 ± 1.32	<0.001	−0.20 ± 0.39	−1.80 ± 1.28	<0.001
Weight, kg	3.78 ± 0.47	3.38 ± 0.51	0.027	6.69 ± 0.53	6.08 ± 0.71	0.013
WAZ	−0.46 ± 0.72	−1.17 ± 0.73	0.028	−0.57 ± 0.62	−1.15 ± 1.07	0.056
Underweight			0.1			0.001
WAZ ≥ −1 SD	78.95	50		83.33	29.41	
WAZ < −1 SD	21.05	50		16.67	70.59	
Length, cm	50.2 ± 2.1	49.1 ± 2.1	0.2	61.57 ± 2.06	58.89 ± 3.06	0.018
LAZ	−1.50 ± 0.98	−2.03 ± 0.78	0.2	−1.40 ± 1.11	−2.33 ± 1.42	0.068
Stunting			0.5			0.1
LAZ ≥ −1 SD	26.32	10		27.78	5.88	
−1 SD > LAZ > −2 SD	42.11	50		44.44	35.29	
LAZ < −2 SD	31.58	40		27.78	58.82	

^a^Mean (SD); n/N (%). ^b^Wilcoxon rank sum test; Wilcoxon rank sum exact test; Fisher’s exact test; Pearson’s Chi-squared test. HCAZ ≥ − 1SD, head-circumference; HCAZ< − 1SD, low or mild impairment in head-circumference.

Infant characteristics by HCAZ are summarized in Table 1. HC measurements differed between infant HCAZ groups. HCAZ differed between infant subgroups at both stages of lactation (*p* < 0.001) and as expected, infant head-circumference was higher in the HCAZ ≥ −1 SD (−0.17 ± 0.53) compared to the HCAZ < −1 SD (−1.43 ± 1.32) in early lactation and in late lactation [HCAZ ≥ −1 SD (−0.20 ± 0.39) vs. HCAZ < −1 SD (−1.80 ± 1.28)]. Weight-for-age-z-score (WAZ, means SD) differed between infants with HCAZ ≥ −1 SD (−0.46 ± 0.72) and those with HCAZ < −1 SD (−1.17 ± 0.73) (*p* = 0.028) but only in early lactation. LAZ did not differ by subgroups at either stage of lactation.

### Human milk microbiome community

**FIGURE 1:**
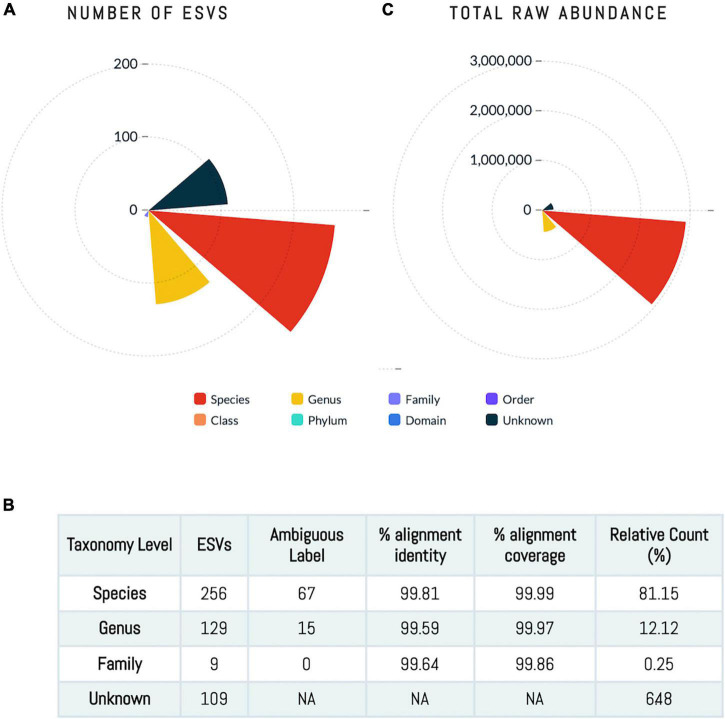
Human milk microbiome community. **(A,C)** The number of ESVs at different taxonomy level showing that sequences annotated at species level account for 81% of the ESVs. **(B)** The total abundance at each taxonomy level.

**FIGURE 2:**
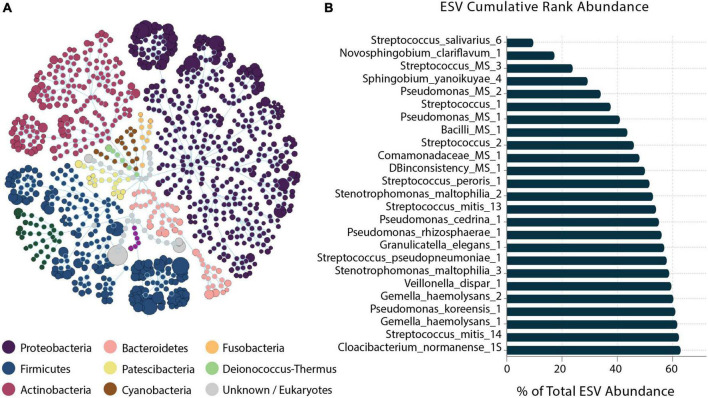
Human milk microbiome community overview. **(A)** The total microbial community colored at phylum level. **(B)** Cumulative abundance of the 25 most abundant ESVs across samples.

A total of 503 ESVs were assembled and captured 3,551,788 sequences reads across all 64 human milk samples (Supplementary File 1). The identified 503 ESVs annotated 256 species (81.2% of reads), 129 genera and 9 family or higher taxa as well as 109 which could not be identified at > 99% similarity (in both identity and coverage) to any known taxa, thus, they were termed Unknowns. Although there were 109 Unknown taxa, these taxa had generally low abundance and contributed to only 6.5% of the total ESVs (Figure 1). The 256 ESVs annotated as putative species had an average BLASTn return for identity at 99.8% and for coverage at 99.99%. At a phyla level, the most prevalent bacteria were from Proteobacteria, followed by Firmicutes, and Actinobacteria. The most abundant species across all samples were *Streptococcus_salivarius_6* and *Novosphingobium_clariflavum_1* contributing to 9.53% and 7.66% of amplicons, respectively. Of the 256 ESVs annotated putative species, 25 taxa comprised 62.84% of the sequenced amplicons (Figure 2). Two of these dominant taxa were the species *Stenotrophomonas_maltophilia*. Although this species was previously isolated from human and raw milk (Spencer, 1995), in the 2004 SENTRY Antimicrobial Surveillance Program among pediatric patients isolates, *Stenotrophomonas_maltophilia* was among the top 15 frequently observed pathogens isolated from North America and Latin America (Fedler et al., 2006; Brooke, 2012). Only one of the 25 dominant taxa, *Pseudomonas_MS_2*, was differentially abundant in the HCAZ ≥ −1 SD in late lactation.

#### Microbial diversity

**FIGURE 3:**
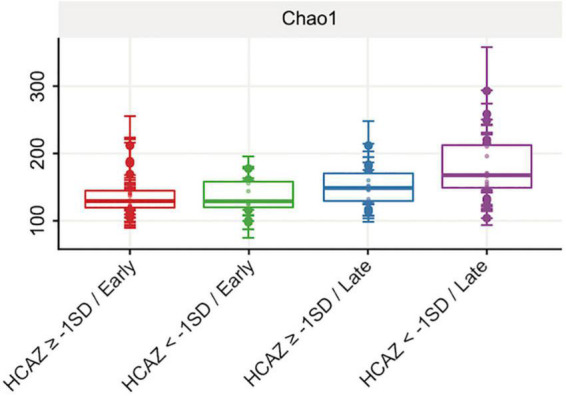
Human milk alpha-diversity (richness) at early and late lactation stages. Comparing head-circumference-for-age-z-score HCAZ ≥ −1 SD (early: *n* = 18; late: *n* = 14) and HCAZ < −1 SD (early: *n* = 11; late: *n* = 19). Alpha diversity Chao1 index was significantly different (*t*-test, *p* = 0.05) between HCAZ ≥ −1 SD and HCAZ< −1 SD at late lactation.

**FIGURE 4:**
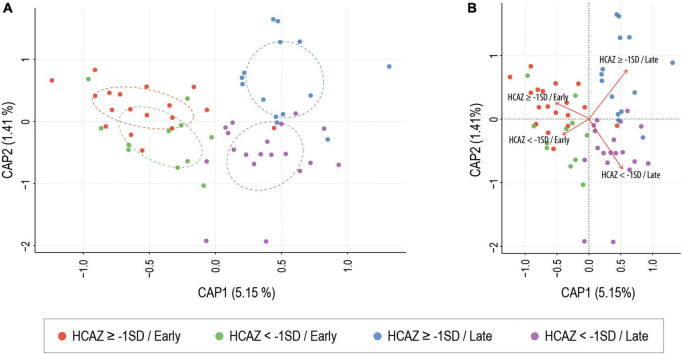
Constrained ordination (CAP) analysis of milk microbial communities present between head-circumference-for-age-z-score (HCAZ ≥ −1 SD) and HCAZ < −1 SD at early and late lactation stages. ANOVA-like permutation test showed significant differences between groups at both stages of lactation (*p* = 0.003). **(A)** Circles represent sample density relative to each group. **(B)** CAP distance biplot showing individual samples colored by groups and vectors (in red) corresponding to the variable loadings (i.e., variable contribution). The black crosses represent the geometric centre of each group.

Of the six alpha-diversity metrices used to measure human milk microbiome communities, only Chao1 index revealed significant differences in microbial community richness between HCAZ ≥ −1 SD and HCAZ < −1 SD in late lactation (*p* = 0.045), but not at early lactation (Figure 3 and Supplementary File 2). Microbial diversity between samples (i.e., beta-diversity) evaluated differences in human milk microbiome communities between the HCAZ ≥ −1 SD and HCAZ < −1 SD at both early and late lactation. Canonical Analysis of Principal Coordinates (CAP) analysis identified significant differences between HCAZ ≥ −1 SD and HCAZ < −1 SD at both stages of lactation (*p* < 0.002) and that also were distinct from one another (Figure 4).

#### Differential abundance in early lactation

**FIGURE 5:**
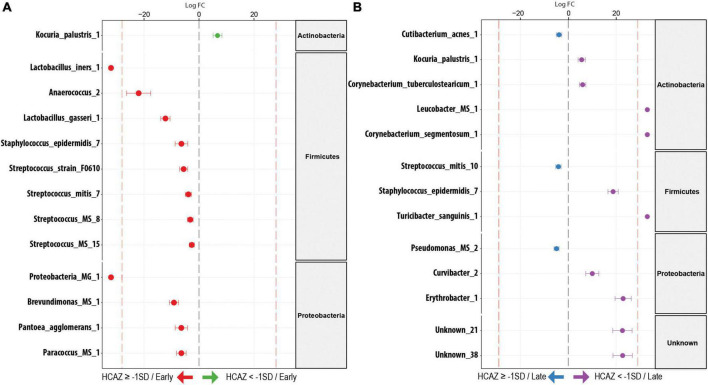
Differentially abundant (DA) ESV associated to head-circumference-for-age-z-score (HCAZ). **(A)** Early lactation: 12 ESVs were significantly more abundant in HCAZ ≥ −1 SD (*n* = 18; left side) and 1 ESV in HCAZ < −1 SD (*n* = 11; right side). **(B)** Late lactation: 3 ESVs significantly more abundant in HCAZ ≥ −1 SD (*n* = 14; left side) and 10 ESVs in HCAZ < −1 SD (*n* = 19; right side). Species are colored and grouped by phylum. The dashed red line represents a limit beyond which ESVs were only quantified in a single group.

During early lactation, DA analysis using DESeq2 identified 13 milk microbiome taxa as significantly different in DA (FDR < 0.05) between HCAZ ≥ –1 SD and HCAZ < −1 SD groups. Interestingly, all except one of the 12 differentially abundant taxa were in the HCAZ ≥ −1 SD, revealing a higher more differentially abundant taxon in the normal HCAZ group. These taxa were annotated as species (12 ESVs) and genera (3 ESVs). The majority of these differentially abundant taxa in the HCAZ ≥ −1 SD group belonged to the Firmicutes genus and included *Lactobacillus_iners_1* (FC = 22.4), *Anaerococcus_2* (FC = 22), *Lactobacillus_gasseri_1* (FC = 12.2), *Staphylococcus_epidermidis_7* (FC = 6.4), *Streptococcus_sp_strain_F0610* (FC = 5.6), *Streptococcus_mitis_7* (FC = 3.9), *Streptococcus_MS_8* (FC = 3.2), and *Streptococcus_MS_15* (FC = 2.6). Proteobacteria had four differentially abundant taxa in the HCAZ ≥ −1 SD group. These were *Proteobacteria_MG_1* (FC = 22.6), *Brevundimonas_MS_1* (FC = 9.1), *Pantoea_agglomerans_1* (FC = 6.4), and *Paracoccus_MS_1* (FC = 6.4). Furthermore, the two taxa; *Proteobacteria_MG_1* and *Lactobacillus_iners_1* were uniquely associated with the HCAZ ≥ −1 SD group. None of the differentially abundant taxa in the HCAZ ≥ −1 SD in early lactation belonged to the Actinobacteria, whereas the only one differentially abundant species *Kocuria_palustris_1* (FC = 6.7) in the HCAZ < −1 SD belonged to the Actinobacteria genus (Figure 5A).

#### Differential abundance in late lactation

In contrast, in late lactation, there was a shift to more differentially abundant species in the HCAZ < −1 SD group. Ten species were differentially abundant in the HCAZ < −1 SD group, revealing more differentially abundant taxa in the infant group with mild impaired HCAZ. Four of these taxa belonged to the Actinobacteria genus including *Kocuria_palustris_1* (FC = 5.6), *Corynebacterium_tuberculostearicum_1* (FC = 6), *Leucobacter_MS* (FC = 22.8), and *Corynebacterium_segmentosum_1* (FC = 23.9). Other differentially abundant species included *Staphylococcus_epidermidis_7* (FC = 18.7) and *Turicibacter_sanguinis_1* (FC = 22.6) from the Firmicutes genus and *Curvibacter_2* (FC = 10) and *Erythrobacter_1* (FC = 23) from the Proteobacteria genus in addition to two Unknown taxa [Unknown_21 (FC = 22.6) and Unknown_38 (FC = 22.7)]. Three differentially abundant species were uniquely associated with HCAZ < −1 SD in late lactation. These species were *Leucobacter_MS, Corynebacterium_segmentosum_1*, and *Turicibacter_sanguinis_1*. On the other hand, only three differentially abundant species were identified in the HCAZ ≥ −1 SD at late lactation. These species were *Cutibacterium_acnes_1* (FC = 3.9), *Pseudomonas_MS_2* (FC = 5), and *Streptococcus_mitis_10* (FC = 4) all of which belong to the Proteobacteria genus (Figure 5B).

## Discussion

Most recent papers that discussed the relationship between human milk and early life brain development had highlighted the role of hormones and neural growth factors (Kim and Yi, 2020) or discussed the role of human milk exposure in promoting cortical indices and improving neurocognitive outcomes (Sullivan et al., 2022). However, there exists a lack of consensus on nutrients in human milk that contribute to brain development (Walfisch et al., 2013; Lockyer et al., 2021). In this study, we explored the association between infant HCAZ and shifts in the human milk microbiome. Six novel findings emerged. First, alpha-diversity differed between HCAZ ≥ −1 SD and HCAZ < −1 SD in late lactation, but not early lactation. Second, beta-diversity based on canonical analysis of principal coordinates identified significant clusters of microbiota by HCAZ at both stages of lactation. Third, in early lactation more differentially abundant species were identified when HCAZ ≥ −1 SD, with most belonging to the Firmicutes genus, but there were two taxa, *Proteobacteria_MG_1* and *Lactobacillus_iners_1* that were uniquely associated with the HCAZ ≥ −1 SD group; in early lactation, only *Kocuria_palustric_1* was differentially association with HCAZ < −1 SD. Fourth, in late lactation, there was a general shift to more differentially abundant species in the HCAZ < −1 SD group, with *Leucobacter_MS*, *Corneybacterium_segnentosum_1*, and *Turicbacter_sanquins_1* uniquely associated with HCAZ < −1 SD. Lastly, there were two Unknown taxa in the HCAZ < −1 SD at late lactation, which may underscore the importance of Unknown taxa and their potential role on infant head growth before 6 months of age. Collectively, our findings show that the HCAZ ≥ −1 SD group had more differentially abundant species of human origin whereas in late lactation the HCAZ < −1 SD group had more differentially abundant environmental species associated with soil, water, and animal sources.

### Diversity of human milk microbiome and head-circumference

The association between milk microbiota and HC growth in early infancy has not been widely explored, however, a few studies have described an association between gut microbiome and HC in premature infants. One study among Indian children living in urban slums reported an association between increased microbial alpha-diversity and greater HC (Huey et al., 2020). Another study in Brazil, compared microbiome diversity in meconium between infants with early HC catch-up growth (<6 months) and infants with late HC catch-up growth (>6 months). They reported greater microbial diversity and a higher abundance of Acinetobacter in meconium from infants with late HC catch-up growth that was independent of infant weight (Terrazzan Nutricionist et al., 2020). Some of these results were consistent with our findings, as we also found HC to be associated with human milk alpha-diversity in late lactation. However, in contrast to both studies, we observed significant differences with beta-diversity between HCAZ infant groups at both stages of lactation as we found more differentially abundant species of the Acinetobacter in the HCAZ ≥ −1 SD group in early lactation.

Some studies corroborate the hypothesis that better neurodevelopment and head growth might be achieved via a favorable gut microbiota. One study found that preterm infants who received a daily dose (10–15 g) of medically graded bee honey (a source of oligosaccharides) had an increased colonization with *Bifidobacterium bifidum* compared to control and this colonization was associated with increased head circumference after 2 weeks (Aly et al., 2017). In randomized, double-blind, placebo-controlled trial of extremely low birth weight infants, supplementation with *Lactobacillus reuteri* promoted HC growth rate during the first month of life (Wejryd et al., 2019). Another study found that after 1 year of supplementation with a symbiotic that included *Lactobacillus* species and fructo-oligosaccharides, the odds of HC < 10% were lower in the supplemented group compared to the control group (Varal et al., 2018). This association between *Lactobacillus* and improved HC is consistent with our findings. In our study at early lactation, *Lactobacillus_gasseri_1* was differentially abundant in the HCAZ ≥ −1 SD group. *Lactobacillus_iners_1* has been associated with probiotic, antioxidant activity, and antimicrobial activity against various pathogens (Gunyakti and Asan-Ozusaglam, 2019) and it was uniquely associated with the HCAZ ≥ −1 SD group in our study.

On the other hand, potentially pathogenic species also have been associated with infant gut microbial composition in preterm infants. Infant gut microbiota that is characterized by a high abundance of Proteobacteria has been associated with “dysbiosis” (Monira et al., 2011; Barrett et al., 2013; Shin et al., 2015). Consistent with our findings, infants with HCAZ ≥ −1 SD had fewer differentially abundant species from Proteobacteria compared to Firmicutes and Actinobacteria. Together, these findings would suggest that specific microbial variations may be associated with HCAZ.

### Differentially abundant taxa and infant head-circumference

DA analyses using DESeq2 identified species that differed between infants with HCAZ ≥ −1 SD group and infants with HCAZ < −1 SD group at both stages of lactation.

#### Early lactation

During early lactation, differentially abundant species in human milk microbiome of infants with the HCAZ ≥ −1 SD were mainly normal human colonizers that included two species belonging to the *Lactobacillus* genus. These two were *Lactobacillus_gasseri_1* and *Lactobacillus_iners_1;* both are common inhabitants of a healthy vaginal flora (Alonzo Martínez et al., 2021). Additionally, *Lactobacillus_gasseri_1* is found in milk and has strains with known probiotic, antioxidant, and antimicrobial activity against various pathogens (Gunyakti and Asan-Ozusaglam, 2019). The second LAB was *Lactobacillus_iners_1*. *Lactobacillus_iners_1* has also been associated with probiotic, antioxidant activity, and antimicrobial activity against various pathogens (Gunyakti and Asan-Ozusaglam, 2019), although some have reported its presence in both healthy and dysbiotic vaginal microbial communities (Macklaim et al., 2013; Marconi et al., 2020; Zheng et al., 2021). *Lactobacillus_iners* is known to have a gram-variable morphology with an unusually small genome (ca. 1 Mbp), suggesting that *Lactobacillus_iners* could have clonal variants associated with both healthy and dysbiotic vaginal environments (Petrova et al., 2017). In our analysis, *Lactobacillus_iners_1* in our human milk samples was uniquely associated with the HCAZ ≥ −1 SD infants supporting our observation of its positive association with increased head circumference in early lactation. Others have also reported that supplementation with members of *Lactobacillus* genera were associated with improved HC during the first month (Wejryd et al., 2019), and lower odds of smaller HC (Varal et al., 2018). Thus, the presence of 2 differentially abundant *Lactobacillus* species in the milk of mothers of infants with HCAZ ≥ −1 SD in early lactation is consistent with these earlier reports and further highlights the importance *Lactobacillus* with improved HC during early lactation.

Other differentially abundant species in the HCAZ ≥ −1 SD group included human commensal bacteria that demonstrated both, protective and stabilizing roles, and potential roles in human infections. These differentially abundant taxa included *Staphylococcus_epidermidis_7*, and *Anaerococcus_2. Staphylococcus_epidermidis* is considered a normal colonizer of human skin and mucous membranes (Otto, 2009). It has been reported as ubiquitous in humans, and as a common inhabitant of healthy human milk environment (Heikkilä and Saris, 2003; Martín et al., 2007) where it may balance the epithelial microflora and serve as a reservoir of resistance genes (Otto, 2009). However, some have described *Staphylococcus_epidermidis* as an opportunistic species (Coates et al., 2014), given that it has been isolated from maternal milk of mothers diagnosed with mastitis (Delgado et al., 2009). Similarly, *Anaerococcus_2*, is an uncharacterized species with several members found in the human vagina, on skin, and in nasal cavities, but it can be involved in human infections (Song et al., 2007; Pépin et al., 2011; Dione et al., 2018). However, human milk is suggested to have the non-virulent strains of these species in early lactation (Soeorg et al., 2017). Researchers have demonstrated that *Staphylococcus_epidermidis* strains genetically similar to those in human milk can replace more virulent strains in the infant gut, suggesting that human milk may play a protective role through the introduction of less-pathogenic strains that could outcompete more virulent strains in the infant gut. This speculation is that gut colonization with virulent strains could be reduced by the mothers’ milk during the first month of life (Soeorg et al., 2013, 2017). This observation suggests the healthy presence of these species acting as early gut colonizers of term infants, and that as commensal bacteria may contribute to early brain growth, as was the case with our HCAZ ≥ −1 SD group.

Other differentially abundant species identified as both normal human colonizers and ambiguous species belonged to the *Streptococcus* genus were also associated with HCAZ ≥ −1 SD. Species previously isolated from the oral cavity and the respiratory tract included *Streptococcus_sp_strain_F0610*, and *Streptococcus_mitis_7*. *Streptococcus_mitis* is recognized as oropharynx bacteria (Kilian et al., 2014) and is found in the infant oral microbiome within few days after birth (Pearce et al., 1995). Two other ambiguous *Streptococcus* taxa were identified. First, *Streptococcus_MS_8* which could represent either *Streptococcus_mitis* or *Streptococcus_pseudopneumoniae*. The second, *Streptococcus_MS_15* which could represent two species, either *Streptococcus_mitis* or *Streptococcus_pneumoniae; Streptococcus_pneumoniae* is one of the leading causes of highly pathogenic infections (Kilian et al., 2014), while *Streptococcus_pseudopneumoniae* is an overlooked pathogen emerging as the causative agent of lower-respiratory-tract infections (Garriss et al., 2019). *Streptococcus_pneumoniae* and *Streptococcus_mitis* represent two opposing lifestyles that have evolved in parallel and can coexist in harmony with their host (Kilian et al., 2014).

The presence of environmental and plant bacteria in human milk have been previously identified and is common (Togo et al., 2019), especially in rural agricultural and hunter-gather communities where there is human interaction with the environment (Blum et al., 2019). Some differentially abundant species in the HCAZ ≥ −1 SD group were environmental species. Among them were the ambiguous taxon *Paracoccus_MS_1. Paracoccus* species include a bright orange color, caused by the synthesis of large amounts of carotenoids (Harker et al., 1998). *Paracoccus_MS_1* was either *Paracoccus_marcusii* or *Paracoccus_carotinifaciens*. *Paracoccus_marcusii* significantly improved the growth, elevated antioxidant property, decreased intestinal permeability, and suppressed the expression of some inflammatory genes in aquatic animal studies (Yang et al., 2015; Xue et al., 2020). It also has probiotic properties (Kalathinathan and Kodiveri Muthukaliannan, 2021), which might explain its presence in milk. Some microbial species in the human milk possess probiotic properties (Dallal et al., 2021), where they utilize the human milk oligosaccharides (Marcobal and Sonnenburg, 2012) resulting in modulation of the infant gut microbiota composition and prevention of pathogen colonization (Bode, 2015). *Paracoccus_carotinifaciens*, which has been also isolated from soil (Tsubokura et al., 1999) produces a carotenoid mixture containing the antioxidant carotenoid astaxanthin (Hayashi et al., 2021) that when supplemented showed protective effects on cognitive function in adults (Hayashi et al., 2018). This functionality and its association with the presence of *Paracoccus* species in the milk of mothers having infants with HCAZ ≥ −1 SD would support a protective functional role in infant brain development. However, this association requires further investigation.

Interestingly, other environmental bacteria were generally considered non-pathogenic; however, they might be opportunistic pathogen for immunocompromised humans. These taxa included: *Brevundimonas_MS_1* and *Pantoea_agglomerans_1. Brevundimonas_MS_1*, is an ambiguous species that could be one of two species, *Brevundimonas_vesicularis* or *Brevundimonas_nasdae*. *Brevundimonas_nasdae* has been reportedly isolated from an Indian lake (Rao and Kumar, 2014). *Brevundimonas_vesicularis* which is ubiquitously found in the environment in soil and water, rarely causes infection in humans; however, it can become an opportunistic pathogen in immunocompromised humans (Ryan and Pembroke, 2018; Stabler et al., 2018). *Pantoea_agglomerans_1* is a plant bacterium that is not a human-pathogen, however, it is also known to cause opportunistic human infections (Cruz et al., 2007; Dutkiewicz et al., 2016). However, the presence of these environmental bacteria in human milk and their associations with infant brain growth during early lactation have never been explored and may require further exploration.

In early lactation, the milk of mothers with infants having HCAZ < −1 SD had only one differentially abundant species; *Kocuria_palustris_1*. *Kocuria* spp. inhabit the normal skin and mucous membrane of the mouth and the digestive and the genital tracts of human and animals (Stackebrandt et al., 1995). However, *Kocuria_palustris_1* was first isolated from cattails in a river tributary (Kovács et al., 1999) and subsequently in rice (Kaga et al., 2009), soil (Caliz et al., 2011), and marine sponges (Martín et al., 2013). Formerly, *Kocuria* spp. were considered non-pathogenic and were rarely associated with human infections (Kandi et al., 2016) but are now considered as pathogenic. Research has highlighted the significance of *Kocuria* in causing infections in pediatric patients (Chen et al., 2015) and they are now being considered as potential pathogens in immunocompromised and pediatric patients (Chen et al., 2015; Moreira et al., 2015). In our study, *Kocuria_palustris_1* was differentially abundant in the HCAZ < −1 SD group at both stages of lactation, indicating an association between *Kocuria_palustris_1* and HCAZ < −1 SD that spans the first 6 months postpartum.

#### Late lactation

The HCAZ ≥ −1 SD group in late lactation had three differentially abundant species. Two of them were normal human colonizers of the skin, oral cavity, gastrointestinal, and genitourinary tract; *Streptococcus_mitis_10* (Pearce et al., 1995; Mitchell, 2011), and *Cutibacterium_acnes_1* (Elston et al., 2019). The third one was the ambiguous species *Pseudomonas_MS_2*, which is one of two soil species *Pseudomonas_putida* (Volke et al., 2020) or *Pseudomonas_hutmensis* (Xiang et al., 2019).

In contrast to the human milk microbiome in early lactation, in late lactation more differentially abundant taxa were associated with infants having HCAZ < −1 SD compared to infants with HCAZ ≥ −1 SD. Several of these taxa were environmental and potentially opportunistic species. As with early lactation, *Kocuria_palustris_1* was identified in the milk of mothers during late lactation and associated with low head circumference at 4–6 months. Other environmental species isolated from aqueous environments included *Curvibacter_2* (Ding and Yokota, 2004, 2010), and *Erythrobacter_1* (Jung et al., 2012; Wu et al., 2012; Li et al., 2017; Yoon, 2017). Moreover, in our study population 2 other environmental species were uniquely associated with the HCAZ < −1 SD group in late lactation; these included *Turicibacter_sanguinis_1* and the ambiguous species *Leucobacter_MS*. *Turicibacter_sanguinis_1* has been isolated from animal feces and skin (Cuív et al., 2011; Maki et al., 2020). *Leucobacter_MS* could be either *Leucobacter_komagatae* or *Leucobacter_aridicollis*. Both have been isolated from contaminated plant and water environments (Morais et al., 2004; Saimmai et al., 2012).

Other differentially abundant species in the HCAZ < −1 SD group were human colonizers with potential pathogenicity. Among them was *Staphylococcus_epidermidis_7*. *Staphylococcus_epidermidis* is a normal human skin and milk colonizers (Heikkilä and Saris, 2003; Martín et al., 2007; Otto, 2009), however, it has been isolated in mastitis cases (Delgado et al., 2009). Although *Staphylococcus_epidermidis* in human milk has the ability to replace virulent strains in the infant gut during early lactation (Soeorg et al., 2013, 2017), this observation has not been examined in the later stage of lactation. Other differentially abundant human colonizers with potential pathogenicity belonged to the genus Corynebacterium which accounts for 30% of the total bacterial inhabitants of human skin (Council et al., 2016). It is a genus with some species that can induce inflammation, skin conditions (Altonsy et al., 2020), and it has been frequently isolated during bovine mastitis (Watts et al., 2001). The first Corynebacterium was *Corynebacterium_tuberculostearicum_1*, which colonizes the human skin and mucosal surfaces and has been frequently isolated from clinical specimens mostly related to the respiratory infections (Hinić et al., 2012) and also has been associated with breast abscesses (Mule et al., 2018) and mastitis (Paviour et al., 2002). The second Corynebacterium species was *Corynebacterium_segmentosum_1*, which was uniquely associated with the HCAZ < −1 SD group in late lactation; it is a gastric microbiota that was suggested, among other gastric species, to negatively affect enteric nervous system modulation via neurogenic inflammatory process (Han et al., 2019). Because the enteric nervous system associated with cognitive function may be regulated by the inflammatory effects of the microbiota (Konturek et al., 2011), the presence of *Corynebacterium_segmentosum_1* in the milk of mothers having HCAZ < −1 SD infants might suggest an association between milk microbial species and head growth through the gut microbiota-brain axis.

In addition, the HCAZ < −1 SD had two differentially abundant ESVs that were poorly characterized at late lactation. These were dominated by unknown sequences, including: TrueUnknown_21 (97% similar to *Prevotella melaninogenica*) and TrueUnknown_38 (98% similar to *Sphingobium yanoikuyae*), which is a soil bacteria (Cunliffe and Kertesz, 2006; Kou et al., 2018). These findings suggest the important potential roles of poorly characterized and unknown bacteria associated with HC, highlighting the substantial amount of knowledge is yet to be discovered regarding the role of human milk microbiome in infant growth and brain development.

## Strengths and limitations

Our study has some limitations. First, the cross-sectional design of our study cannot establish causation between the human milk microbiome and infant HCAZ. Second, this study might be under-powered. Third, due to the lack of studies that associate the human milk microbiome with infant growth parameters, the exploratory nature of this study, and the potential significant biological role of some species despite their low abundance, low-count taxa were included in our study, but require further investigation. Fourth, we used the eubacteria primers 27F/533R. Although 27F/533R primers are often used in human milk studies and have high coverage of “core” genera including, *Cutibacterium* (Hunt et al., 2011; Jost et al., 2013; Jiménez et al., 2015; Lackey et al., 2019; Gonzalez et al., 2021), it might not amplify species within the *Bifidobacterium*, which is a “core” human milk genus (Klindworth et al., 2013) but there is insufficient sequence variation to discriminate between closely related taxa (Johnson et al., 2019). Known inconsistencies do exist in the perceived “core” genera of human milk microbiota commonly identified in healthy mothers (Jeurink et al., 2013; Fitzstevens et al., 2017).

Despite these inconsistencies, V1–V3 sequencing has produced comparable results to the full-length 16S rRNA V1–V9 in the human gut microbiome samples at species-level and was shown to be highly informative when used in conjunction with an appropriate identity threshold (Johnson et al., 2019). In our study, we used the ANCHOR pipeline, which provided high-resolution analysis for species-level microbial identification (Gonzalez et al., 2019) in conjunction with > 99% identity and coverage threshold. Thus, ESV-based approaches may potentially resolve species-level diversity in the human milk microbiome, when compared to higher level annotation (i.e., phyla, family, or genera level) (Johnson et al., 2019), making it a strength of our study. However, we understand that, because many species remain poorly characterized and mistakes exist in major repositories, species should be considered putative even when single species sequences share 100% 16S rRNA gene fragment similarity (Gonzalez et al., 2019, 2021). In addition, given the milk microbiome community changes by the stage of lactation (Gonzalez et al., 2021) and our study was of a cross-sectional design, the milk sample collections at two points of lactation allowed us to establish the association between milk the microbiome and the infant HCAZ. The homogeneity of our cohort might have been an asset and because our healthy mothers did not have sub-clinical mastitis and did not take antibiotics, we minimized the effect on the milk microbiome ecosystem. However, we cannot infer if the milk microbiome was the only determinant factor for the HC growth.

## Conclusion

In conclusion, we observed that the milk microbiome of a cohort of unrelated Guatemalan mothers was associated with infant HCAZ at both early and late lactation. DESeq2 identified a total of 26 differentially abundant species between HCAZ ≥ −1 SD and HCAZ < −1 SD during early and late lactation. In early lactation, the HCAZ ≥ −1 SD group had more differentially abundant *Streptococcus* species, with several human colonizers. However, there were some potentially pathogenic species that emerged. In late lactation, the HCAZ < −1 SD group had more differentially abundant species, with several of them of environmental origin or having potential pathogenicity. To our knowledge, this is the first study to explore the association between the human milk microbiome and infant HCAZ during lactation. These insights collectively highlight need to continue to investigate the role of milk microbiome in infant growth.

## Data availability statement

Publicly available datasets were analyzed in this study. This data can be found here: European Genome-Phenome Archive (EGAD00001004160).

## Ethics statement

McGill Institutional Review Board and CeSSIAM Human Subjects Committee reviewed and approved the studies involving human participants. All participating mothers provided written informed consent for participation.

## Author contributions

TA drafted the manuscript. TA and EG performed the statistical analyses. EG analyzed the microbiome data and created the figures. NS provided the funding and supervised the field data collection. KK provided the funding for the 16s RNA analysis and framed with TA the experimental design. All authors contributed to the article and approved the submitted version.

## Supplementary material

Supplementary File 1 A total of 503 ESVs were assembled and captured 3,551,788 sequences reads across all 64 human milk samples. The identified 503 ESVs annotated 256 species (81.2% of reads), 129 genera and 9 family or higher taxa as well as 109 which could not be identified at > 99% similarity (in both identity and coverage) to any known taxa, thus, they were termed Unknowns.

Supplementary File 2 Six alpha-diversity metrices used to measure human milk microbiome communities, only Chao1 index revealed significant differences in microbial community richness between HCAZ ≥ −1 SD and HCAZ < −1 SD in late lactation (*p* = 0.045).

## Abbreviations

DA: Differentially Abundant
HC: Head-circumference
HCAZ: Head-Circumference-for-Age-z-Score
LAZ: Length-for-Age-z-Score
WAZ: Weight-for-Age-z-Score.
